# Topics of the Month

**Published:** 1896-10

**Authors:** 


### TOPICS OF THE MONTH.

THE opening of the new Riverside Hospital, 306 Lafayette avenue, on Wednesday evening, September 16, 1896, was an interesting event in the history of this well-established institution. Dr. Randall, assisted by the hospital staff, were busy receiving and all had a pleasant word for the comfortably arranged, airy, well-lighted and cozy home, which would be more proper than to call it a hospital. Twenty-five patients can be cared for at one time and on the opening evening twelve beds were occupied. A cordial invitation is extended to all physicians to call and inspect the hospital.

---

ATTENTION is called on another page to the twenty-ninth annual meeting of the Medical Association of Central New York, to be held in Rochester, October 20th next. This association is next to the state societies in point of numbers, work and influence, and should be liberally attended by the Buffalo profession. The number of Buffalo members is far too small as compared with the Syracuse or Rochester membership, and it is to be hoped that a large number of applicants may attend the Rochester meeting.

---

THE Mexican delegates at the recent meeting of the Public Health Association attracted considerable attention both by the secular press and by local visitors to the meetings. Their prompt attendance, active participation and intense interest shown in the papers and discussions, showed them to be scientific hygienists of superior merit. The delegation consisted of the health officer of each of the several states, and it was but fitting that their superior, or permanent president of the superior board of health, of which they are all members, should also be the president of the Buffalo meeting.

---

EFFECTS OF THE PUBLIC HEALTH ASSOCIATION.

We have boiled the hydrant water,
We have sterilised the milk ;
We have strained the prowling microbe
Through the finest kind of silk ;
We have bought and we have borrowed
Every patent health device,
And at last the doctor tells us
That we've got to boil the ice.

—Unknown.

